# The Role of Metabolic Disorders and Laboratory Abnormalities in Wound Healing and Recovery in Geriatric and Non-Geriatric Orthopedic Patients in Poland—Prospective Research

**DOI:** 10.3390/jcm14155317

**Published:** 2025-07-28

**Authors:** Robert Węgłowski, Bartosz Borowski, Anna Bronikowska, Piotr Piech, Grzegorz Staśkiewicz, Jaromir Jarecki

**Affiliations:** 1 Department of Correct, Clinical and Imaging Anatomy, Medical University of Lublin, Chodźki 4 (CSM), 20-093 Lublin, Polandbronikowska.ania@gmail.com (A.B.);; 2 Department of Orthopedics and Traumatology, Medical University of Lublin, Jaczewskiego 8, 20-090 Lublin, Poland; jaromir.jarecki@umlub.pl; 3 Department of Radiology, Medical University of Lublin, Jaczewskiego 8, 20-090 Lublin, Poland

**Keywords:** orthopedic patients, prospective research, Poland, patient well-being, wound healing

## Abstract

**Objectives:** This study sought to assess the impact of diabetes and hypertension on wound healing and recovery in orthopedic patients, with an emphasis on laboratory correlations. **Materials and Methods:** This study included 67 orthopedic patients, divided into a geriatric group (n = 49, ≥65 years) and a control group (n = 18). Clinical and laboratory assessments were performed at admission and discharge. Data were analyzed statistically. **Results:** Geriatric patients showed a higher triglyceride glucose-body mass index (TyG-BMI), glucose, cholesterol, C-reactive protein (CRP), interleukin-6 (IL-6), and leukocytes and lower hemoglobin and platelets (PLTs), with poorer healing and well-being. Elevated CRP, IL-6, and urea and decreased protein and hemoglobin persisted in this group. Diabetes improved outcomes in older adults, while hypertension worsened them in younger patients. Favorable outcomes correlated with higher triglycerides, fibrinogen, hemoglobin, and red blood cells (RBCs), while they did not correlate with elevated CRP, IL-6, leptin, urea, creatinine, and white blood cells (WBCs). **Conclusions:** Key predictors of healing and well-being included CRP, hemoglobin, RBC, and hematocrit in older patients and hypertension, CRP, hemoglobin, and leptin in younger individuals. Age-specific metabolic and inflammatory profiles influence recovery trajectories and may be used to predict problems in both recovery and patients’ well-being. Further research is required to better understand the correlations between these factors.

## 1. Introduction

The wound healing process and postoperative recovery following orthopedic procedures present a significant clinical challenge, particularly in patients with metabolic disorders. In both elderly and younger individuals, the presence of chronic conditions such as obesity or diabetes can markedly delay tissue regeneration, increase the risk of infection, and hinder the return to full functional capacity [1]. In addition to chronic diseases, aging itself negatively affects tissue repair and recovery. One of the most important age-related mechanisms is immunosenescence, a gradual decline in immune system function, including reduced naive T-cell production, low-grade chronic inflammation (inflammaging), and impaired coordination of immune responses. These changes contribute to delayed wound healing and prolonged inflammatory phases in elderly patients, even in the absence of comorbidities [2]. These complications in the therapeutic process can lengthen a patient’s stay at the hospital and thus generate greater costs for the health system. In recent years, there has been growing interest in the wound healing process, driven in part by population aging, the increasing prevalence of diabetes, and persistent disparities in access to medical care [3]. Some research strongly suggests the implementation of personalized nutritional plans, as well as interdisciplinary care for the elderly, as it increases their quality of life and promotes healing [4]. Many important factors are commonly underestimated and neglected, such as the need for an adequate mattress due to the poor mobility of geriatric patients, or a proper dietary approach [5]; therefore, it is important to assess the quality of health system facilities in terms of providing the most up-to-date, comprehensive care for patients [6]. Another equally important factor impairing tissue repair processes is arterial hypertension, which is one of the most common health disorders in the world. Chronically elevated blood pressure leads to microcirculatory damage and impaired tissue perfusion, thereby reducing the availability of oxygen and essential nutrients required for regeneration [7]. Interestingly, certain antihypertensive medications—particularly calcium channel blockers—have shown potential in supporting wound healing, partly by enhancing tissue perfusion and stimulating fibroblast proliferation [8]. Laboratory parameters are playing an increasingly important role in assessing the stages of wound healing. Neutrophils are involved in the inflammatory response accompanying the healing process; however, their excessive activation may lead to secondary tissue damage and chronic inflammation. Pro-inflammatory cytokines—interleukin-1 (IL-1), IL-6, and tumor necrosis factor (TNF-α)—in turn, significantly influence neutrophil activity by modulating their phagocytic capacity [9]. Monitoring laboratory parameters can provide valuable prognostic insights and guide therapeutic decision-making, improving patients’ well-being and speeding up their discharge. The primary objectives of this study were to determine the impact of selected metabolic disorders, such as diabetes and arterial hypertension, on wound healing and the recovery process in orthopedic patients, and to analyze correlations between specific laboratory parameters, wound healing outcomes, and patient well-being. The secondary objective was to compare these associations between geriatric and non-geriatric populations, in order to identify potential differences in impaired regenerative processes and risk factors for the delayed recovery of patients.

## 2. Materials and Methods

This prospective study included 67 patients hospitalized for orthopedic reasons at a district hospital in eastern Poland. Patients’ data were gathered from December 2023 to December 2024. All patients were admitted to the orthopedic ward due to musculoskeletal injuries resulting in either fractures of the upper or lower limb bones or ligamentous injuries of one of the joints. Table 1 presents a detailed breakdown of the reasons for orthopedic ward admission.

The study group consisted of geriatric patients (49 individuals), while the control group comprised younger, non-geriatric patients (18 individuals). The cut-off age value for both groups was 65 years old, as patients aged 65 or more were incorporated into the study group, and below this value, to the control group. Participants ranged in age from 38 to 98 years. The mean age was 73.57 years, and the median age was 75 years. Among the 67 patients enrolled, there were 38 women and 29 men.

Each participant was asked to complete an informed consent form prior to inclusion in this study. After obtaining consent and enrolling the patients in the project, basic demographic and clinical data were collected from all participants, including age, sex, height, body weight, waist circumference, and blood pressure. In addition, a medical history was obtained to assess comorbid conditions. Laboratory tests were performed using peripheral blood samples, and cardiovascular risk was evaluated using the SCORE or SCORE-OP scale. Based on physician interviews, the patients’ general well-being, postoperative wound healing, and overall recovery were assessed using a numeric rating scale from 1 to 10. The wound assessment was performed by various physicians, with the use of specially selected, author-made criteria, in order to minimize the possibility of human errors. These were not validated clinical scales but structured subjective evaluations intended for internal clinical use. Laboratory parameters, as well as well-being and recovery scores, were recorded twice—at the beginning and at the end of hospitalization, to be precise—on the day of a patient’s admission and their discharge from the ward.

Exclusion criteria were defined for this study, including the presence of congenital metabolic disorders that could distort the results relative to the general population—such as storage diseases—as well as any issues beyond the research team’s control that prevented the collection of repeated laboratory measurements and clinical assessments.

After completing the database of included patients, statistical analysis was performed using R statistical software (A Language and Environment for Statistical Computing, version 4.4.2). Categorical variables were presented with n (%). Numerical variables were presented with the mean ± SD or median (interquartile range), depending on the normality of distribution. The normality of distribution was assessed with the Shapiro–Wilk test, skewness, and kurtosis. Variance homogeneity was assessed with Levene’s test. For comparisons of independent groups, Student’s *t* test, Welch’s *t* test, the Mann–Whitney U test, Pearson’s chi-square test, or Fisher’s exact test was used, as appropriate. For parameter comparisons between the first and second samples, a paired *t*-test and Wilcoxon test were used, as appropriate. For identifying significant relationships between well-being and healing assessment with blood parameters, Spearman correlation analysis was used. All statistical calculations assumed an *alpha* = 0.05.

This study was approved by the bioethics committee of the local medical university (number of the opinion: KE-0254/255/12/2023, date: 14 December 2023). The committee found no violations of generally accepted bioethical principles governing scientific research.

It is worth emphasizing that approximately 75% of the literature cited in this study was published within the last five years, reflecting a modern and innovative investigation of the research problem and ensuring this study’s relevance and up-to-date character.

## 3. Results

Initially, data and results obtained during the first assessment were compared between the study group and the control group. The analysis focused on differences in general patient characteristics (age, weight, waist circumference, etc.), laboratory test results, and information gathered during physician interviews. This comparison is presented in Table 2 and Table 3.

Based on the presented results, the study group demonstrated statistically significant differences compared to the control group, including a higher TyG-BMI index (*p* = 0.018), glucose level (*p* = 0.001), total cholesterol (*p* = 0.018), CRP (*p* = 0.001), IL-6 (*p* = 0.001), and leukocyte count (*p* = 0.001). Additionally, significantly lower levels of hemoglobin (*p* < 0.001) and PLT (*p* < 0.001) were observed. Statistically significantly lower levels of well-being and poorer wound healing outcomes were observed in the study group compared to the control group (MD = −3.00, 95% CI [–3.00; −1.00], *p* = 0.002, and MD = −2.00, 95% CI [–2.00; 0.00], *p* = 0.021, respectively). Additionally, the SCORE percentage was significantly higher among geriatric patients than among younger individuals (*p* = 0.002). The distribution of high versus very high SCORE categories differed significantly between the geriatric and non-geriatric groups (*p* = 0.004). Notably, no patients in the geriatric group had a high SCORE, whereas in the younger group, 22.2% (n = 4) fell into the high-risk category. The proportion of patients with a very high SCORE was greater in the geriatric group compared to the younger group (100.0% vs. 77.8%).

In the next step, laboratory parameters, as well as assessments of well-being and wound healing, were compared between the two groups based on the results and information obtained during the second measurement and physician evaluation. The comparison is presented in Table 4.

Statistical analysis of the results allows for the formulation of the following statements (only statistically significant associations are reported):

CRP levels were significantly higher among geriatric patients compared to younger patients (MD = 7.10, 95% CI [2.20; 15.20], *p* = 0.001).

Total protein levels were significantly lower in the geriatric group compared to the non-geriatric group (MD = −0.39, 95% CI [−0.59; −0.18], *p* < 0.001).

IL-6 levels were significantly higher among geriatric patients compared to younger patients (MD = 39.18, 95% CI [5.47; 46.07], *p* < 0.001).

Urea levels were also significantly elevated in the geriatric group (MD = 7.00, 95% CI [6.00; 10.00], *p* = 0.001).

Conversely, hemoglobin levels were significantly lower in geriatric patients compared to the younger group (MD = −1.25, 95% CI [–1.60; −0.60], *p* = 0.001).

Subsequently, the impact of selected components of the metabolic syndrome on patient well-being and wound healing was analyzed. The first component examined was diabetes. The results are presented in Table 5.

In the geriatric group, a significant association was found between patient-reported well-being during the first visit and the presence of diabetes. Patients with diabetes reported higher well-being compared to those without diabetes (MD = 1.06, 95% CI [0.22; 1.90], *p* = 0.015).

Additionally, a significant association was observed between physician-assessed wound healing during the second visit and diabetes in the geriatric group. Higher healing scores were noted among diabetic patients compared to non-diabetic patients (MD = 0.73, 95% CI [0.03; 1.42], *p* = 0.040).

No significant relationships, between diabetes and well-being or wound healing assessments, were identified in the younger group (*p* > 0.05).

The second component of the metabolic syndrome examined was arterial hypertension. The results of the statistical analysis are presented in Table 6.

In the younger age group, a significant association was observed between the presence of hypertension and patient-reported well-being during both visit 1 and visit 2. Patients with hypertension reported lower well-being scores compared to those without hypertension (MD = −3.00, 95% CI [−3.00; −1.00], *p* = 0.007, and MD = −2.00, 95% CI [−3.00; −1.00], *p* = 0.002, respectively).

In the younger age group, a significant association was also found between the presence of hypertension and physician-assessed wound healing during both visit 1 and visit 2. Patients with hypertension had lower healing scores compared to those without hypertension (MD = −2.30, 95% CI [−3.67; −0.93], *p* = 0.003, and MD = −1.38, 95% CI [−2.22; −0.53], *p* = 0.003, respectively).

No significant relationship between hypertension and assessments of well-being or wound healing was found in the geriatric group (*p* > 0.05).

The final step was to determine whether there was an association between specific laboratory parameters and the level of patient well-being or the quality of wound healing in both groups. The relationships between the analyzed outcomes and laboratory markers are presented in Table 7.

### 3.1. Patient Well-Being and Laboratory Results

In both age groups, a moderate positive correlation was observed between general well-being and cholesterol (visit 1), fibrinogen (visit 1), and PLT (visit 1). This indicates that higher levels of these parameters were moderately associated with higher well-being scores across both groups.

A moderate to high positive correlation was observed between general well-being and triglycerides (visit 1), fibrinogen (visit 2), and hemoglobin (visit 1), indicating that higher levels of these parameters were moderately to strongly associated with better well-being in both age groups.

Conversely, a moderate to high negative correlation was found between general well-being and CRP levels (both visits), suggesting that higher CRP concentrations were moderately to strongly associated with lower well-being assessments across both groups.

Exclusively in the geriatric group, a moderate positive correlation was observed between general well-being and triglycerides (visit 2), albumin (both visits), total protein (visit 2), hemoglobin (visit 2), and PLT (visit 2). This indicates that higher levels of these parameters were moderately associated with higher well-being assessments within the study group.

A moderate to high positive correlation was observed between general well-being and potassium (both visits), RBC (both visits), and hematocrit (both visits), indicating that higher levels of these parameters were moderately to strongly associated with better well-being in the geriatric group.

A moderate negative correlation was found between general well-being and creatinine (both visits), IL-6, (visit 1), urea (both visits), and WBC (visit 1), suggesting that higher levels of these markers were moderately associated with lower well-being assessments in the research group.

Exclusively in the younger patient group, a moderate negative correlation was observed between general well-being and both CRP (visit 2) and IL-6 (visit 2), indicating that higher levels of these inflammatory markers were moderately associated with lower well-being assessments in the control group.

A strong negative correlation was found between general well-being and leptin levels (both visits), suggesting that elevated leptin concentrations were highly associated with poorer well-being in the control group.

### 3.2. Wound Healing and Laboratory Results

In both age groups, a moderate positive correlation was observed between the wound healing process and triglycerides (visit 2), albumin (visit 2), fibrinogen (visit 1), and PLT (visit 1), indicating that higher levels of these parameters were moderately associated with improved healing assessments.

A moderate to high positive correlation was also found between wound healing and triglycerides (visit 1), fibrinogen (visit 2), hemoglobin (visit 1), and hematocrit (visit 2), suggesting that elevated levels of these markers were moderately to strongly associated with better wound healing outcomes in both groups.

A moderate to high negative correlation was observed between the wound healing process and CRP (visit 1) as well as leptin (visit 1), indicating that higher levels of these parameters were moderately to strongly associated with poorer healing outcomes in both groups.

Exclusively in the geriatric group, a moderate positive correlation was found between wound healing and glucose (visit 1), albumin (visit 1), and PLT (visit 2), suggesting that higher levels of these markers were moderately associated with improved healing assessments in this population.

A moderate to high positive correlation was observed between the wound healing process and potassium levels (both visits), indicating that higher potassium concentrations were moderately to strongly associated with better healing outcomes in the geriatric group.

A strong positive correlation was found between wound healing and hemoglobin (visit 2), RBC (both visits), and hematocrit (visit 1), suggesting that elevated levels of these parameters were highly associated with improved healing assessments in the geriatric group.

Conversely, a moderate negative correlation was observed between wound healing and creatinine (both visits), urea (both visits), and WBC (visit 1), indicating that higher levels of these markers were moderately associated with poorer healing outcomes in the research group.

Exclusively in the younger group, a moderate positive correlation was observed between the wound healing process and cholesterol (visit 1), indicating that higher cholesterol levels were moderately associated with better healing assessments in the control group.

Moderate negative correlations were found between wound healing and glucose (visit 1), IL-6 (visit 2), leptin (visit 2), and WBC (visit 1), suggesting that elevated levels of these parameters were moderately associated with poorer wound healing outcomes in the control group.

## 4. Discussion

Based on the analysis of the obtained results, it can be concluded that the presence of diabetes or arterial hypertension affects both well-being and the wound healing process within specific patient groups. Additionally, the following laboratory parameters were shown to influence these outcomes: cholesterol, fibrinogen, PLT, triglycerides, hemoglobin, CRP, albumin, total protein, potassium, RBC, hematocrit, IL-6, WBC, urea, glucose, and leptin.

### 4.1. Diabetes

This study demonstrated that among geriatric patients, those with a diagnosis of diabetes reported better well-being and more favorable wound healing outcomes compared to non-diabetic patients. However, it is important to note that none of the diabetic patients in this study had poorly controlled diabetes—the highest blood glucose level recorded in the geriatric group during the initial assessment was 123 mg/dL. An additional explanation for this association may lie in the fact that diabetic patients are often treated with medications aimed at minimizing the disease’s adverse effects and are typically well-educated about the potential complications of unmanaged diabetes. Muijs et al. point out in their study that there is currently no consensus regarding the relationship between blood glucose levels and patient mood [10]. However, it is important to consider the concept of “diabetes-related distress” (DRD), defined as a general decline in well-being caused by the daily challenges of living with diabetes [11]. Factors such as smoking and marital status have also been shown to influence the overall severity of DRD [12].

Patients with diabetes often experience impaired wound healing, a phenomenon with multifactorial etiology. Contributing factors include hyperglycemia, neuropathy, hypoxia, vascular damage, and increased susceptibility to bacterial infections [13]. Prolonged hyperglycemia leads to the generation of reactive oxygen species, oxidative stress, and excessive activation of autophagy and apoptosis pathways, all of which negatively impact the wound healing process [14].

To more accurately investigate the relationship between diabetes, patient well-being, and wound healing in individuals hospitalized for orthopedic reasons, future studies should be conducted on a larger population that includes patients with varying levels of glycemic control. Nonetheless, it is worth remembering the implications that diabetes may cause, while investigating patients suffering from this condition.

### 4.2. Arterial Hypertension

The next component of the metabolic syndrome analyzed in this study was arterial hypertension. Statistical analysis revealed that the presence of hypertension was associated with lower well-being scores among younger patients (control group). However, no such correlation was observed in the geriatric population (study group). This finding may be explained by the fact that most older individuals with hypertension are already aware of their condition due to routine screenings or blood pressure measurements conducted during visits to primary care physicians. Such diagnosed hypertension is typically well-controlled with pharmacological treatment, which helps prevent complications associated with elevated blood pressure. However, according to Kawabe et al., in Japan, approximately 8% of men and 1% of women under the age of 40 are diagnosed with hypertension [15]. Furthermore, a study by Yano et al. [16] emphasizes the seriousness of this diagnosis in young adults, reporting that among 4851 participants, 2574 had normal blood pressure readings, while the remaining individuals were classified as having elevated blood pressure or various stages of hypertension. Notably, only 4% of all participants were receiving antihypertensive medication [16]. Carey et al. [17] report that young adults are less frequently diagnosed with hypertension, have poorer blood pressure control, and tend to be less concerned about the condition. This attitude is influenced by various factors, including reluctance to “admit” being ill, concerns about the safety of antihypertensive medications, and the perceived financial burden of long-term treatment [17].

The impact of hypertension on mental well-being has been explored by Ang et al., who suggest that patients with uncontrolled hypertension are more likely to develop mental health disorders compared to those with controlled hypertension [18]. Similarly, Särnholm et al. [19] propose that the lower mood observed in hypertensive patients may stem from anxiety about potential complications, such as stroke or acute coronary syndrome. However, the authors emphasize that appropriate patient counseling and education about the condition can significantly mitigate the negative effects on psychological well-being [19].

In addition to its impact on well-being, statistical analysis revealed a negative effect of arterial hypertension on wound healing among younger patients. The detrimental influence of elevated blood pressure on regenerative processes is partly due to impaired angiogenesis and reduced vascular reactivity, resulting in poorer perfusion of healing tissues [20]. Beyond impairing tissue regeneration, hypertension may also lead to abnormal healing patterns, such as the formation of keloids [21]. It is also important to note that untreated hypertension is a key contributor to the development of atherosclerosis. Li et al. highlight that the biochemical cascade triggered by atherosclerosis impairs wound healing [22]. Moreover, sclerotic, narrowed vessels exhibit reduced blood flow, thereby limiting the delivery of essential nutrients and oxygen to regenerating tissues. As arterial hypertension is one of the most common abnormalities globally, it is crucial to investigate its influence on the mood and postoperative reconvalescense.

### 4.3. Total Cholesterol

After analyzing the impact of selected components of metabolic syndrome, laboratory parameters were examined. The first of these was the total cholesterol concentration. Statistical analysis revealed that higher cholesterol levels correlated with better well-being in both geriatric and non-geriatric patients. However, it should be noted that in both groups, the highest cholesterol concentrations were only slightly above the accepted laboratory reference range (249 mg/dL in the geriatric group and 222 mg/dL in the younger group), and the overall sample size was relatively small.

To date, few studies have directly investigated the influence of cholesterol levels on mood and mood disorders. Jones et al. suggest several potential mechanisms underlying this association, including microvascular pathologies in the central nervous system, impaired cholesterol metabolism in the brain, and disturbances in gut microbiota [23]. Regarding the latter, depressive disorders have been linked to the overgrowth of Enterococcus species, which promote the development of chronic systemic inflammation [24].

It is important to recognize that the relationship between cholesterol levels and mood may be bidirectional. Patients experiencing mood disorders, such as depression, often neglect their diet and may engage in chronic substance use, which can lead to abnormalities in laboratory findings, including disturbances in lipid profiles [25].

Statistical analysis also revealed that higher cholesterol levels were correlated with improved wound healing among non-geriatric patients. However, it is important to consider the previously mentioned limitations of this study—namely, the relatively small sample size and cholesterol concentrations that deviated only slightly from the accepted reference ranges.

Qin et al. explain that elevated cholesterol levels, as observed in certain conditions such as diabetes, can induce endothelial cell apoptosis and impair wound healing [26]. Moreover, increased cholesterol levels promote its accumulation in macrophages involved in tissue regeneration. These cholesterol-laden macrophages exhibit reduced motility and efficiency, and they may also deposit within vessel walls, contributing to atherosclerotic plaque formation, which further hinders the wound healing process [27,28].

### 4.4. Fibrinogen

The next laboratory parameter examined was fibrinogen. Analysis showed that higher fibrinogen levels were correlated with better well-being in both age groups, as well as with improved wound healing. It is noteworthy that all measured fibrinogen values remained within the accepted reference range. The observed correlation can be interpreted in light of fibrinogen’s role in tissue repair, as it serves as a precursor to fibrin—the primary structural component of the hemostatic clot. As emphasized by Zuliani-Alvarez and Midwood, the globular FBG domains present in the β and ɣ chains of fibrinogen contribute to the formation of the fibrin fiber network, whose structure and stability directly influence the dynamics of tissue repair. Moreover, fibrinogen modulates the inflammatory response through interactions with leukocyte integrins, facilitating wound site clearance and promoting regeneration. The authors highlight that even within the reference range, subtle variations in fibrinogen levels may have significant implications for both hemostatic processes and subsequent phases of tissue remodeling [29].

In our study, a positive correlation was observed between fibrinogen levels and patient-reported well-being, which may appear contradictory to previous findings in the literature. It is important to emphasize that the relatively small size of the study group may affect the stability of the observed correlations. Additionally, in prior research, fibrinogen has often been analyzed as a marker of inflammation associated with lower psychological well-being [30], which may help explain the discrepancies between our results and those reported in earlier studies.

### 4.5. Platelets

Among the laboratory parameters analyzed, both in geriatric and non-geriatric patients, higher PLT showed a positive correlation with better self-reported well-being and more effective wound healing. It is important to note that all PLT values remained within the reference range, and only in the non-geriatric group did they exceed 300,000/μL. According to the literature, platelets play a crucial role not only in hemostasis but also in the initiation and progression of wound healing processes. As described by Rodrigues et al. [7], immediately following tissue injury, platelets become activated, accumulate at the injury site, and release the contents of their granules—including platelet-derived growth factor (PDGF), transforming growth factor-β (TGF-β), and vascular endothelial growth factor (VEGF)—thereby initiating a local inflammatory response and promoting tissue regeneration. The released mediators stimulate fibroblast proliferation, myofibroblast differentiation, and angiogenesis. Furthermore, interactions between platelets and immune cells play a critical role in regulating the inflammatory process, which in turn affects the rate and quality of wound healing [7]. A normal platelet count in patients reflects physiological activation; however, lower values within the reference range may be associated with less effective wound healing. The clinical application of platelet function is increasingly evident in modern practice, particularly through the use of autologous platelet-rich plasma (PRP) in wound treatment, due to the ability of platelets to release bioactive factors that support tissue regeneration and remodeling [31].

According to our analysis, higher PLT levels were positively correlated with patient well-being. He et al. emphasized that beyond their hemostatic function, platelets also exhibit neurobiological activity through the storage and release of serotonin—a key neurotransmitter involved in mood regulation [32]. These findings suggest the potential utility of the platelet count as a marker for both wound healing efficiency and psychophysical well-being.

### 4.6. Triglycerides

Another laboratory parameter analyzed in this study was the triglyceride concentration. Statistical analysis showed that higher triglyceride levels correlated with better well-being and improved wound healing in both age groups. However, similar to total cholesterol, the highest triglyceride level recorded in this study was 178 mg/dL, which does not significantly exceed widely accepted laboratory reference ranges. Additionally, the relatively small sample size limits the generalizability of these correlations.

According to Wei et al., a high triglyceride-glucose index (TyG) in older adults is associated with a poorer cognitive function [33]. The European Atherosclerosis Society also emphasizes that elevated triglyceride levels are a risk factor for conditions such as atherosclerosis and stroke [34]. In their study, Sara et al. highlight that endothelial dysfunction—an early marker of atherosclerosis—is correlated with depressive episodes [35]. Additionally, cerebral atherosclerosis may lead to ischemia in brain regions involved in mood and emotional regulation. The resulting ischemia-induced inflammation can damage deep layers of the white matter, potentially leading to symptoms such as apathy [36]. Therefore, it can be concluded that triglycerides, although not directly, may influence patients’ mood—especially when reaching pathologically high levels.

The impact of elevated triglyceride levels on wound healing can be considered analogous to the previously described correlation between cholesterol and this process. As noted earlier, the formation of atherosclerotic plaque—promoted by abnormal triglyceride levels—leads to impaired vascular flow, which ultimately compromises tissue healing.

### 4.7. Hemoglobin

Another parameter evaluated for its impact on both wound healing and patient well-being was hemoglobin. A strong positive correlation was found between higher hemoglobin levels and more favorable outcomes in both endpoints, regardless of age group. These findings are supported by the study conducted by Haddad et al. [37], which investigated the relationship between admission hemoglobin levels and mortality risk following femoral neck fracture surgery.

This retrospective study, involving 626 elderly patients, demonstrated that lower hemoglobin levels were significantly associated with higher six-month mortality. In multivariate analysis, hemoglobin was identified as an independent prognostic factor (*p* = 0.007), and a cutoff value of 10.45 g/dL was established as predictive of mortality risk [37]. These findings confirm that anemia can negatively affect the course of recovery and the wound healing process, emphasizing the importance of early diagnosis and correction of preoperative deficiencies in this patient population.

Moreover, the use of hemoglobin sprays for hard-to-heal wounds is gaining popularity in contemporary clinical practice. Elg and Hunt conducted a detailed evaluation of the effectiveness of hemoglobin spray as an adjunctive therapy in the treatment of postoperative wounds. Its application resulted in a more than 2.7-fold increase in the weekly probability of wound healing compared to standard care alone (*p* = 0.001). Importantly, the therapeutic benefit of hemoglobin spray was evident as early as the first week of treatment. Within four weeks, an average wound area reduction of 102% greater than that observed in the control group was reported. Additionally, patients with postoperative wounds experienced significantly less pain [38], indicating improved well-being—an outcome that aligns with the findings observed in our study. As hemoglobin is part of a standard blood tests in all hospitalized patients, its importance as a healing efficiency predictor is crucial in clinical practice.

### 4.8. C-Reactive Protein

CRP is one of the most sensitive markers of the inflammatory response in the human body. Our analysis demonstrated that elevated CRP levels were significantly associated with delayed wound healing and poorer patient-reported well-being in both age groups.

In the study by Pinchuk et al. [39], a significant relationship was observed between elevated CRP levels and impaired postoperative wound healing. The average postoperative CRP level in patients who developed wound infections was 10.4 mg/L—substantially higher than in the group without such complications, where the average value was 4.8 mg/L. At the same time, the authors emphasize that CRP should not be regarded as an independent predictive factor; however, its elevated level constitutes a significant risk indicator for impaired healing processes [39].

Elevated serum CRP levels have also been significantly associated with mood disorders such as depression and bipolar disorder, suggesting a negative impact of active inflammation on patients’ overall well-being [40]. As one of the basic laboratory tests, CRP stands out as a cheap, globally available predictor of inflammation, and thus prolonged tissue healing.

### 4.9. Albumin Level

Statistical analysis revealed that higher albumin levels were correlated with better well-being in geriatric patients, as well as improved wound healing in both age groups. Al Marwani et al. reported that the likelihood of developing depression was significantly lower in patients with higher albumin levels compared to those with lower levels [41].

The plasma albumin concentration is often considered an indicator of nutritional status and overall physiological well-being and has been shown to closely correlate with patients’ quality of life (QoL) [42]. Naga Rohith et al. also identified a relationship between lower preoperative albumin levels and prolonged hospitalization, as well as an increased incidence of wound infections and wound dehiscence [43]. Furthermore, albumins complexed with basic fibroblast growth factor (HSA-bFGF) are used as agents to support wound healing [44].

### 4.10. Total Protein

Analysis revealed a significant positive correlation between total protein levels and improved well-being in the geriatric patient group. This association was not observed in the non-geriatric group, nor was a significant correlation found between total protein levels and wound healing.

A study by Yin et al. [45] demonstrated that reduced serum total protein (TP) levels were significantly correlated with the severity of depressive episodes in patients diagnosed with schizophrenia. These findings support the hypothesis that total protein—as a nonspecific marker of nutritional status and acute-phase activation—may have not only diagnostic but also prognostic value in the context of patient well-being and mental functioning [45].

Although some reports suggest a relationship between total protein levels in wound exudate and the wound’s healing potential, most available data are based on studies conducted in very small patient cohorts, limiting the ability to draw definitive conclusions. Further large-scale clinical studies are needed to evaluate the utility of total protein as a potential indicator of the wound healing process.

### 4.11. Potassium

In the geriatric population, a significant positive correlation was observed between serum potassium levels and both the effectiveness of wound healing and overall patient well-being. No such association was found in the non-geriatric group.

Currently available studies do not provide conclusive evidence of a direct correlation between serum potassium levels and wound healing or patient well-being. However, scientific evidence suggests that both dietary potassium intake and systemic potassium levels may influence mental health—contributing to reductions in depressive symptoms, decreased tension, and improvements in vitality and subjective well-being [46].

### 4.12. Erythrocytes and Hematocrit

In the age-stratified analysis, both the RBC count and hematocrit levels showed a positive correlation with wound healing, particularly in the geriatric population. The RBC count was additionally associated with improved patient-reported well-being in this group. Recent scientific reports have increasingly highlighted the role of erythrocytes in hemostasis and tissue repair. It has been demonstrated that RBCs contribute to clot formation and contraction through interactions with platelets, resulting in the creation of a densely packed structure composed of polyhedral erythrocytes. This organized architecture forms an almost impermeable barrier that protects the injury site and promotes tissue regeneration [47].

As hematocrit is a reflection of erythrocyte concentration in the blood, it influences the rheological properties of blood, including viscosity and microcirculatory flow. These factors directly affect oxygen and nutrient delivery to damaged tissues, thereby playing a crucial role in the wound healing process. In our study, hematocrit levels were positively correlated with wound healing in both age groups, while an association with improved well-being was observed only in geriatric patients. Excessively low hematocrit levels may impair perfusion and contribute to hemostatic disturbances and delayed tissue repair. These findings suggest that hematocrit may serve not only as a marker of hematologic status but also as a potential therapeutic target in managing wound healing and coagulation-related complications [48].

While direct studies linking RBC or hematocrit levels with subjective well-being remain limited, the present data emphasize their joint relevance in assessing recovery in orthopedic patients.

### 4.13. Interleukin 6

Another laboratory parameter analyzed was IL-6—a pro-inflammatory cytokine that plays a key role in the body’s inflammatory and immune response. In our analysis, a significant correlation was observed between elevated IL-6 levels and poorer self-reported well-being in both geriatric and non-geriatric patient groups.

In the study by Foley et al. [49], IL-6 activity was shown to correlate significantly with subjective well-being in patients with depression. Notably, IL-6 was particularly associated with aspects of well-being related to physical discomfort and fatigue, rather than cognitive or anxiety-related symptoms [49]. Moderate levels of IL-6 support the proper progression of the proliferative phase of wound healing, in part by stimulating fibroblast activity and promoting angiogenesis. However, excessively high or prolonged elevation of IL-6—commonly observed in chronic inflammatory states—can disrupt granulation tissue formation and impair tissue remodeling. As a result, this may lead to delayed wound healing, the development of chronic wounds, or pathological scarring, such as hypertrophic scars and keloids [50].

### 4.14. Leukocytes

The analysis included the total WBC count as a nonspecific marker of the inflammatory response. In the geriatric patient group, a significant negative correlation was observed between higher WBC levels and lower self-reported well-being. Importantly, in both age groups—geriatric and non-geriatric—an elevated WBC count was associated with delayed wound healing.

The literature data suggest that higher WBC counts—even within the reference range—may adversely affect cognitive well-being in older individuals. The study by Li et al. [51] demonstrated that elevated WBC levels—even within the normal range—were significantly associated with cognitive decline. Individuals with cognitive impairment had higher WBC counts than cognitively intact individuals (*p* < 0.05), and this relationship remained significant after adjusting for confounding factors [51].

Leukocytes, particularly neutrophils, play a critical role in initiating the wound healing process by clearing debris and eliminating pathogens. However, a persistently elevated WBC count, especially in the context of a prolonged inflammatory phase, may disrupt the transition to the proliferative phase and result in delayed wound healing [52].

It should be noted that our dataset included only total WBC counts. Differential leukocyte data—such as neutrophil, eosinophil, and lymphocyte percentages—were not available, which limits the ability to assess the contribution of specific immune cell types to the observed healing outcomes.

### 4.15. Urea

One of the analyzed parameters was urea. Higher urea levels were significantly correlated with reduced self-reported well-being and delayed wound healing in the geriatric patient group. These associations were not observed in the non-geriatric group.

A study based on data from the China Health and Retirement Longitudinal Study (CHARLS) demonstrated that an elevated blood urea nitrogen-to-creatinine ratio (BUNCr) is significantly associated with poorer cognitive functioning and more severe depressive symptoms. These findings highlight the importance of monitoring urea levels not only as a marker of renal function [53].

Elevated urea concentrations negatively affect cell migration, proliferation, and differentiation, ultimately impairing the proliferative phase and disrupting tissue remodeling during wound healing. Additionally, uremia weakens the immune response and promotes chronic inflammation, increasing the risk of infections and the persistence of chronic wounds [54].

### 4.16. Glucose

Statistical analysis did not reveal a significant association between glucose levels and patient well-being in either of the studied groups. However, it is worth noting that disturbances in glucose metabolism are more frequently observed in individuals with psychiatric disorders involving mood dysregulation, such as schizophrenia and bipolar disorder [55]. Moreover, a study by Lui et al. found that both excessively low and high fasting glucose levels were associated with an increased risk of suicide, highlighting the potential role of glycemic imbalance in mental health outcomes [56].

Regarding wound healing, this study found that higher glucose levels were correlated with better healing outcomes among geriatric patients, whereas the opposite trend was observed in the non-geriatric population—lower blood glucose levels were associated with more effective wound healing. It is important to note, however, that glucose measurements in this study did not indicate any substantial deviations from widely accepted reference ranges. Among geriatric patients, the highest recorded glucose level was 123 mg/dL, while in the younger group, it was 100 mg/dL. The absence of markedly elevated values may be attributed to several factors, including the clinical requirement that diabetic patients scheduled for surgery should demonstrate adequate glycemic control prior to the procedure. It should also be noted that the relatively small group of non-geriatric patients did not include individuals with diabetes. It is well-established that poorly regulated blood glucose levels negatively affect the body’s healing processes, particularly in cases of uncontrolled diabetes, where complications such as microangiopathy and peripheral neuropathy frequently occur.

At the cellular level, impaired tissue healing is influenced by factors such as excessive cytokine production, elevated levels of reactive oxygen species, and molecular mechanisms that promote the presence of pro-inflammatory M1 macrophages [57]. One of the more modern approaches to enhancing wound healing in diabetes involves modulating macrophage polarization—shifting from the inflammation-promoting M1 phenotype to the regeneration-supporting M2 phenotype [58].

### 4.17. Leptin

Statistical analysis revealed a significantly negative impact of elevated leptin levels on the well-being of patients in the non-geriatric group. Additionally, a strong inverse correlation between leptin concentration and wound healing efficiency was observed in both age groups.

It is important to note that the relatively small sample size may limit the reliability of these correlations, and leptin levels in all study participants remained within reference ranges. The literature remains divided regarding the role of leptin in mood regulation and neuropsychiatric disorders. Cao et al. reported that among individuals over the age of 40 with a BMI > 25, those experiencing a depressive episode had higher leptin levels compared to healthy controls [59]. In contrast, the study by Zou et al. suggests a beneficial role of leptin in cognitive processes and mood regulation [60].

These conflicting findings highlight the need for more rigorously conducted clinical studies on large patient cohorts to clarify the relationship between leptin levels and mood, as well as its potential involvement in psychiatric and neurodegenerative disorders.

Numerous studies also suggest a beneficial role of leptin in the wound healing process. Yuan et al. highlight leptin’s involvement in modulating the inflammatory response, promoting angiogenesis and re-epithelialization, and supporting fibroblast proliferation and differentiation [61]. However, the authors emphasize that the specific role of leptin in each of these processes warrants further investigation [61]. Another study, conducted by Yiğittürk et al. [62], demonstrated that leptin can accelerate wound healing by enhancing endothelial nitric oxide synthase (eNOS) expression and improving endothelial cell viability in an in vitro wound model. The authors observed that this effect was dose-dependent, with the highest wound closure rate (93.7%) occurring at a leptin concentration of 100 ng/mL. These findings suggest a significant role for leptin in promoting angiogenesis and tissue regeneration [62].

## 5. Conclusions

To conclude, among the analyzed disorders and laboratory parameters, the greatest impact on well-being in the geriatric population appears to be associated with CRP, hemoglobin, red blood cell count, and hematocrit. In contrast, in the non-geriatric group, well-being was most strongly influenced by the presence of arterial hypertension, as well as levels of CRP, hemoglobin, and leptin.

Regarding the effectiveness of the wound healing process, the most significant contributing factors in the geriatric group were CRP, hemoglobin, red blood cell count, hematocrit, and leptin. In the non-geriatric group, the key determinants were the presence of hypertension, along with CRP, hemoglobin, hematocrit, and leptin levels.

Given the limitations of this study and the inconsistencies and gaps in the available literature, there is a clear need for comprehensive and well-designed research aimed at elucidating the precise relationships between the analyzed laboratory parameters, metabolic disorders, and both well-being and wound healing outcomes in patients across different age groups.

Obtaining definitive insights into these associations could contribute to shorter hospital stays, thereby reducing the burden on healthcare systems, while simultaneously improving patient well-being and accelerating recovery.

## 6. Limitations and Strengths

This study had several limitations. One of the primary limitations was the small sample size in both the study and control groups, with the sample size relative to the number of potential predictors insufficient to provide a robust and generalizable regression model, leaving this work only based on correlations. Another limitation stemmed from the involvement of more than one physician in patient assessment, which—despite adherence to a standardized evaluation protocol—may have introduced minor discrepancies in the assessment of well-being and wound healing across both patient groups. Furthermore, all patients included in this study were recruited from a single center in eastern Poland and shared a similar ethnic background. As a result, the findings may not be directly generalizable to more diverse populations or different geographical regions. A final limitation was the relatively uniform values of the analyzed laboratory parameters, which often did not significantly deviate from commonly accepted reference ranges. Despite the several limitations, this study carries a general message to bear in mind the associations and collocations between laboratory parameters, metabolic disorders, patients’ recovery process, and that some of the analyzed parameters may prove useful in predicting the efficiency of this process. This study included a prospective and uniform data collection protocol, the use of clinically meaningful endpoints such as wound healing and subjective well-being, and the simultaneous assessment of multiple metabolic and hematologic parameters. The homogeneity of the patient cohort in terms of diagnosis and treatment pathway increases the internal validity of the findings and allowed for a focused analysis of recovery-related predictors.

## Figures and Tables

**Table 1:** Reasons for hospitalization in the study and control groups.

Diagnosis	Study Group (n = 49)	Control Group (n = 18)
Distal radius fracture	7	3
Intertrochanteric femoral fracture	14	2
Femoral neck fracture	14	1
Lower leg fracture (excluding lateral tibial condyle fracture)	7	4
Lateral tibial condyle fracture	6	2
Anterior cruciate ligament (ACL) injury	0	4
Wrist bone fracture	0	1
Phalangeal fracture	0	1

**Table 2:** Baseline patient characteristics collected during the initial assessment—demographic and clinical data.

Variable	Geriatric Patients (n = 49)	Younger Patients (n = 18)	MD (95% CI)	*p*
Age [years]	80.00 ± 9.35	56.06 ± 10.58	23.94 (18.61; 29.28)	<0.001 ^1^
Sex				
Female	30 (61.2)	8 (44.4)	-	0.342 ^2^
Male	19 (38.8)	10 (55.6)		
Injury location				
Leg	42 (85.7)	13 (72.2)	-	0.281 ^3^
Arm	7 (14.3)	5 (27.8)		
Height [cm]	168.00 (163.00; 175.00)	173.00 (168.00; 179.50)	−5.00 (−8.00; 0.00)	0.106
Weight [kg]	75.00 (70.00; 88.00)	75.00 (70.00; 75.00)	0.00 (−2.00; 10.00)	0.183
Waist [cm]	94.00 (86.00; 98.00)	88.00 (86.00; 90.00)	6.00 (0.00; 10.00)	0.045
BMI	26.57 (22.86; 33.12)	25.06 (21.73; 26.57)	1.51 (−3.71; 11.39)	<0.05
TyG-BMI	248.37 (209.54; 269.53)	206.74 (206.23; 234.81)	41.63 (2.79; 42.35)	0.018
Diabetes				
Yes	21 (42.9)	4 (22.2)	-	0.207 ^2^
No	28 (57.1)	14 (77.8)		
Diabetes—type *				
I	7 (33.3)	1 (25.0)	-	>0.999 ^3^
II	14 (6–6.7)	3 (75.0)		
Nicotine				
Yes	23 (46.9)	9 (50.0)	-	>0.999 ^2^
No	26 (53.1)	9 (50.0)		
General well-being (patient) [1–10]	2.00 (2.00; 4.00)	5.00 (3.00; 6.00)	−3.00 (−3.00; −1.00)	0.002
Healing assessment (doctor) [1–10]	3.00 (2.00; 5.00)	5.00 (3.00; 6.00)	−2.00 (−2.00; 0.00)	0.021
Metabolic syndrome				
Yes	36 (73.5)	10 (55.6)	-	0.270 ^2^
No	13 (26.5)	8 (44.4)		
SCORE [%]	0.30 (0.20; 0.38)	0.12 (0.10; 0.26)	0.18 (0.05; 0.19)	0.002
SCORE				
High	0 (0.0)	4 (22.2)	-	0.004 ^3^
Very high	49 (100.0)	14 (77.8)		

Data presented as n (%) for categorical variables and mean ± SD or median (interquartile range) for numeric variables, depending on the distribution. Groups compared with Student’s *t* test ^1^, the Mann–Whitney U test, Pearson’s chi-square test ^2^, or Fisher’s exact test ^3^, as appropriate. * Aggregates the number of patients with diabetes. MD—mean or median difference (geriatric vs. younger); 95% CI—95% confidence interval; *p*—probability; TyG-BMI—triglyceride glucose-body mass index; SCORE—systematic coronary risk evaluation.

**Table 3:** Baseline patient characteristics collected during the initial assessment—laboratory data.

Variable	Geriatric Patients (n = 49)	Younger Patients (n = 18)	MD (95% CI)	*p*
Glucose [mg/dL]	100.00 (91.00; 123.00)	90.00 (86.00; 100.00)	10.00 (5.00; 22.00)	0.007
HDL [mg/dL]	46.34 ±14.71	54.00 ± 10.11	−7.66 (−15.17; −0.14)	0.046 ^1^
LDL [mg/dL]	85.00 (75.30; 103.00)	73.20 (60.80; 83.80)	11.80 (2.10; 21.00)	0.010
Cholesterol [mg/dL]	218.94 ± 30.05	200.11 ± 21.92	18.83 (3.33; 34.32)	0.018 ^1^
Triglycerides [mg/dL]	158.00 (145.00; 165.00)	160.00 (151.25; 171.00)	−2.00 (−15.00; 4.00)	0.393
CRP [mg/L]	9.60 (5.90; 12.10)	1.75 (0.92; 6.77)	7.85 (3.60; 8.80)	0.001
Albumin [g/dL]	4.60 (4.00; 5.00)	5.00 (4.60; 5.10)	−0.40 (−0.60; 0.00)	0.011
Protein [g/dL]	6.90 (6.30; 6.90)	6.90 (6.80; 6.97)	0.00 (−0.20; 0.00)	0.313
Creatinine [mg/dL]	1.00 (0.80; 1.20)	0.85 (0.80; 0.98)	0.15 (0.00; 0.20)	0.030
Fibrinogen [mg/dL]	288.00 (253.00; 305.00)	278.00 (253.00; 288.00)	10.00 (−5.00; 35.00)	0.122
Il-6 [pg/mL]	16.20 (12.80; 41.35)	6.90 (2.10; 12.88)	9.30 (5.10; 16.16)	0.001
Leptin [ng/dL]	19.30 (13.20; 34.45)	13.05 (9.80; 78.14)	6.25 (−7.91; 9.00)	0.399
Sodium [mmol/L]	136.00 (135.00; 138.00)	137.00 (136.00; 137.00)	−1.00 (−2.00; 0.00)	0.110
Potassium [mmol/L]	4.00 (3.30; 4.10)	4.00 (3.70; 4.10)	0.00 (−0.40; 0.10)	0.567
Urea [mg/dL]	40.00 (36.00; 58.00)	35.00 (26.75; 41.00)	5.00 (0.00; 15.00)	0.038
Hemoglobin [g/dL]	12.10 (10.20; 12.80)	13.10 (12.83; 13.10)	−1.00 (−1.50; −0.30)	<0.001
RBC [×mln/µL]	4.00 (3.30; 4.10)	4.10 (4.00; 4.10)	−0.10 (−0.50; 0.00)	0.116
Hematocrit [%]	38.20 (32.30; 39.20)	38.20 (31.20; 39.20)	0.00 (−1.00; 1.10)	0.670
WBC [×thsnd/μL]	8.90 (8.00; 10.20)	7.60 (6.80; 8.55)	1.30 (0.60; 2.10)	0.001
PLT [×thsnd/μL]	273.00 (202.00; 293.00)	298.00 (276.00; 302.00)	−25.00 (−40.00; −8.00)	<0.001

MD—mean or median difference (geriatric vs. younger); 95% CI—95% confidence interval; *p*—probability; HDL—high-density lipoprotein; LDL—low-density lipoprotein; CRP—C-reactive protein; Il-6—interleukin 6; RBC—red blood cells; WBL—whole blood lymphocytes; PLT—platelets. Groups compared with Student’s *t* test ^1^.

**Table 4:** Well-being, wound healing, and laboratory parameters collected during the second assessment.

Variable	Geriatric Patients (n = 49)	Younger Patients (n = 18)	MD (95% CI)	*p*
General well-being (patient) [1–10]	5.00 (5.00; 7.00)	7.00 (5.00; 7.00)	−2.00 (−2.00; 0.00)	0.119
Healing assessment (doctor) [1–10]	7.00 (6.00; 7.00)	7.00 (6.00; 8.00)	0.00 (−1.00; 0.00)	0.128
Glucose [mg/dL]	93.00 (90.00; 100.00)	82.00 (75.00; 90.75)	11.00 (3.00; 16.00)	0.006
HDL [mg/dL]	42.00 (34.00; 58.00)	50.00 (46.00; 56.00)	−8.00 (−12.00; 0.00)	0.077
LDL [mg/dL]	80.00 (78.20; 105.00)	71.30 (65.00; 91.00)	8.70 (6.80; 24.00)	0.006
Cholesterol [mg/dL]	208.00 (200.00; 232.00)	200.00 (190.00; 205.00)	8.00 (0.00; 26.00)	0.034
Triglycerides [mg/dL]	155.10 ± 23.15	152.28 ± 15.99	2.82 (−9.02; 14.67)	0.635 ^1^
CRP [mg/L]	12.10 (7.20; 30.20)	5.00 (4.10; 9.28)	7.10 (2.20; 15.20)	0.001
Albumin [g/dL]	4.10 (3.90; 4.80)	4.65 (4.20; 5.00)	−0.55 (−0.80; −0.20)	0.003
Protein [g/dL]	6.25 ± 0.38	6.64 ± 0.36	−0.39 (−0.59; −0.18)	<0.001 ^1^
Creatinine [mg/dL]	0.80 (0.70; 1.00)	0.70 (0.70; 0.80)	0.10 (0.00; 0.20)	0.055
Fibrinogen [mg/dL]	319.51 ± 52.72	314.06 ± 53.25	5.45 (−23.64; 34.55)	0.709 ^1^
IL-6 [pg/mL]	52.30 (19.30; 68.20)	13.12 (9.92; 24.55)	39.18 (5.47; 46.07)	<0.001
Leptin [ng/dL]	23.20 (13.80; 70.30)	39.75 (38.20; 88.32)	−16.55 (−26.40; 0.00)	0.065
Sodium [mmol/L]	137.00 (137.00; 138.00)	138.50 (138.00; 139.00)	−1.50 (−2.00; 0.00)	0.008
Potassium [mmol/L]	3.93 ± 0.24	4.18 ± 0.43	−0.25 (−0.41; −0.08)	0.004 ^1^
Urea [mg/dL]	38.00 (38.00; 40.00)	31.00 (28.50; 32.00)	7.00 (6.00; 10.00)	0.001
Hemoglobin [g/dL]	11.60 (11.20; 12.10)	12.85 (12.65; 13.10)	−1.25 (−1.60; −0.60)	0.001
RBC [×mln/µL]	3.80 (3.60; 3.90)	3.90 (3.60; 4.07)	−0.10 (−0.30; 0.00)	0.110
Hematocrit [%]	35.20 (32.00; 38.20)	39.20 (37.52; 39.88)	−4.00 (−4.90; −0.90)	0.016
WBC [×thsnd/μL]	9.34 ± 1.48	8.46 ± 1.45	0.88 (0.07; 1.69)	0.033 ^1^
PLT [×thsnd/μL]	247.92 ± 50.42	268.78 ± 28.72	−20.86 (−40.68; −1.04)	0.040 ^2^

Calculations based on visit 2. Data presented as mean ± SD or median (interquartile range) for numeric variables, depending on distribution. MD—mean or median difference (geriatric vs. younger). Groups compared with Student’s *t* test ^1^, Welch’s *t* test ^2^, or the Mann–Whitney U test, as appropriate.

**Table 5:** Association between the presence of diabetes and patient well-being and wound healing.

Variable	Geriatric Patients				Younger Patients			
	Diabetes		MD (95% CI)	*p*	Diabetes		MD (95% CI)	*p*
	Yes (n = 21)	No (n = 28)			Yes (n = 4)	No (n = 14)		
Visit 1								
General well-being (patient) [1–10]	3.67 ± 1.28	2.61 ± 1.57	1.06 (0.22; 1.90)	0.015 ^1^	3.50 (2.00; 5.25)	5.00 (3.25; 6.00)	−1.50 (−4.00; 1.00)	0.380
Healing assessment (doctor) [1–10]	5.00 (3.00; 6.00)	3.00 (2.00; 4.00)	2.00 (0.00; 2.00)	0.0504	3.50 (2.00; 5.25)	5.00 (3.25; 6.75)	−1.50 (−4.00; 1.00)	0.194
Visit 2								
General well-being (patient) [1–10]	5.00 (5.00; 7.00)	5.00 (4.75; 7.00)	0.00 (−1.00; 1.00)	0.856	5.00 (5.00; 5.75)	7.00 (5.25; 7.00)	−2.00 (−2.00; 1.00)	0.731
Healing assessment (doctor) [1–10]	6.76 ± 0.94	6.04 ± 1.35	0.73 (0.03; 1.42)	0.040 ^1^	6.50 (5.00; 8.00)	7.00 (6.00; 8.00)	−0.50 (−3.00; 2.00)	0.616

Data presented as mean ± SD or median (interquartile range), depending on distribution. MD—mean or median difference (yes vs. no). Groups compared with Student’s *t* test ^1^ or the Mann–Whitney U test, as appropriate.

**Table 6:** Association between the presence of arterial hypertension and patient well-being and wound healing.

Variable	Geriatric Patients				Younger Patients			
	Hypertension		MD (95% CI)	*p*	Hypertension		MD (95% CI)	*p*
	Yes (n = 21)	No (n = 28)			Yes (n = 8)	No (n = 10)		
Visit 1								
General well-being (patient) [1–10]	4.00 (2.00; 5.00)	2.00 (2.00; 4.00)	2.00 (0.00; 2.00)	0.092	3.00 (2.75; 4.25)	6.00 (5.00; 6.00)	−3.00 (−3.00; −1.00)	0.007
Healing assessment (doctor) [1–10]	4.00 (2.00; 5.00)	3.00 (2.00; 6.00)	1.00 (−1.00; 1.00)	0.909	3.50 ± 1.41	5.80 ± 1.32	−2.30 (−3.67; −0.93)	0.003 ^1^
Visit 2								
General well-being (patient) [1–10]	5.10 ± 1.04	5.71 ± 1.44	−0.62 (−1.36; 0.13)	0.102 ^1^	5.00 (4.00; 5.25)	7.00 (7.00; 7.00)	−2.00 (−3.00; −1.00)	0.002
Healing assessment (doctor) [1–10]	6.00 (5.00; 7.00)	7.00 (6.00; 7.00)	−1.00 (−1.00; 0.00)	0.348	6.12 ± 0.99	7.50 ± 0.71	−1.38 (−2.22; −0.53)	0.003 ^1^

Data presented as mean ± SD or median (interquartile range), depending on distribution. MD—mean or median difference (yes vs. no). Groups compared with Student’s *t* test ^1^ or the Mann–Whitney U test, as appropriate.

**Table 7:** Association between patient well-being and wound healing and selected laboratory parameters.

Variable	Correlation with General Well-Being (Patient) [1–10]								Correlation with Healing Assessment (Doctor) [1–10]							
	Visit 1				Visit 2				Visit 1				Visit 2			
	Geriatric Patients		Younger Patients		Geriatric Patients		Younger Patients		Geriatric Patients		Younger Patients		Geriatric Patients		Younger Patients	
	rho	*p*	rho	*p*	rho	*p*	rho	*p*	rho	*p*	rho	*p*	rho	*p*	rho	*p*
Glucose [mg/dL]	0.19	0.179	−0.44	0.065	0.17	0.256	−0.14	0.566	0.45	0.001	−0.50	0.033	0.16	0.268	−0.44	0.064
HDL [mg/dL]	−0.27	0.058	−0.27	0.283	0.08	0.608	−0.09	0.737	−0.19	0.182	−0.23	0.358	0.08	0.589	−0.29	0.235
LDL [mg/dL]	0.26	0.074	−0.19	0.461	0.01	0.925	−0.01	0.972	0.22	0.137	−0.11	0.651	0.14	0.344	0.04	0.867
Cholesterol [mg/dL]	0.42	0.003	0.49	0.040	0.01	0.954	0.28	0.262	0.27	0.064	0.59	0.010	0.09	0.553	0.32	0.196
Triglycerides [mg/dL]	0.56	<0.001	0.75	<0.001	0.52	<0.001	0.39	0.105	0.44	0.002	0.81	<0.001	0.40	0.005	0.64	0.005
CRP [mg/L]	−0.35	0.013	−0.76	<0.001	−0.13	0.383	−0.52	0.027	−0.35	0.013	−0.83	<0.001	−0.12	0.415	−0.44	0.064
Albumin [g/dL]	0.29	0.044	0.09	0.711	0.46	0.001	0.18	0.470	0.35	0.015	0.02	0.929	0.59	<0.001	0.52	0.026
Protein [g/dL]	0.12	0.430	−0.38	0.125	0.43	0.002	0.36	0.142	0.23	0.110	−0.51	0.031	0.29	0.044	0.55	0.017
Creatinine [mg/dL]	−0.65	<0.001	−0.05	0.834	−0.51	<0.001	−0.23	0.367	−0.49	<0.001	−0.02	0.942	−0.51	<0.001	−0.14	0.587
Fibrinogen [mg/dL]	0.41	0.004	0.51	0.031	0.54	<0.001	0.76	<0.001	0.51	<0.001	0.53	0.024	0.44	0.001	0.79	<0.001
Il-6 [pg/mL]	−0.35	0.013	−0.26	0.297	0.03	0.860	−0.66	0.003	−0.20	0.177	−0.25	0.320	0.28	0.055	−0.51	0.030
Leptin [ng/dL]	−0.10	0.480	−0.81	<0.001	−0.04	0.807	−0.71	0.001	−0.50	<0.001	−0.79	<0.001	−0.15	0.294	−0.67	0.002
Sodium [mmol/L]	0.06	0.695	0.09	0.735	0.20	0.176	−0.19	0.455	0.16	0.278	0.00	0.986	−0.02	0.915	−0.07	0.790
Potassium [mmol/L]	0.70	<0.001	0.18	0.475	0.59	<0.001	−0.11	0.664	0.80	<0.001	0.11	0.670	0.42	0.003	0.11	0.652
Urea [mg/dL]	−0.62	<0.001	0.06	0.798	−0.52	<0.001	−0.12	0.624	−0.60	<0.001	0.24	0.343	−0.46	0.001	−0.17	0.511
Hemoglobin [g/dL]	0.70	<0.001	0.69	0.001	0.58	<0.001	0.32	0.197	0.83	<0.001	0.63	0.005	0.74	<0.001	0.22	0.375
RBC [×mln/µL]	0.76	<0.001	0.17	0.511	0.63	<0.001	0.01	0.958	0.81	<0.001	−0.05	0.846	0.70	<0.001	0.09	0.713
Hematocrit [%]	0.75	<0.001	0.03	0.897	0.44	0.002	0.19	0.441	0.91	<0.001	−0.12	0.634	0.52	<0.001	0.78	<0.001
WBC [×thsnd/μL]	−0.54	<0.001	−0.45	0.061	−0.04	0.766	−0.38	0.116	−0.56	<0.001	−0.46	0.058	−0.15	0.320	−0.64	0.005
PLT [×thsnd/μL]	0.41	0.004	0.62	0.006	0.45	0.001	−0.07	0.779	0.58	<0.001	0.68	0.002	0.35	0.015	0.15	0.542

rho—Spearman correlation coefficient.

## Data Availability

Any data will be provided by the authors if requested.
